# Remote Activation of Mechanotransduction via Integrin Alpha-5 via Aptamer-Conjugated Magnetic Nanoparticles Promotes Osteogenesis

**DOI:** 10.3390/pharmaceutics16010021

**Published:** 2023-12-22

**Authors:** Hadi Hajiali, Michael Rotherham, Alicia J. El Haj

**Affiliations:** Healthcare Technologies Institute, Institute of Translational Medicine, School of Chemical Engineering, University of Birmingham, Birmingham B15 2TH, UK

**Keywords:** magnetic nanoparticles, integrin alpha-5, aptamer, mechanotransduction, osteogenesis, bone regeneration

## Abstract

Bone regeneration and repair are complex processes in the adult skeleton, and current research has focused on understanding and controlling these processes. Magnetic nanoparticle (MNP)-based platforms have shown potential in tissue engineering and regenerative medicine through the use of magnetic nanomaterials combined with remotely applied dynamic fields. Previous studies have demonstrated the ability of MNP-induced mechanoactivation to trigger downstream signaling and promote new bone formation. In this study, we aimed to compare the osteogenic induction achieved using the mechanoreceptor targets, Piezo1, Fzd1, Fzd2, and integrin alpha-5. We compared the binding efficacy of different types of agonists (antibodies vs. aptamers) to these receptors. Moreover, we optimized the aptamer concentration (2.5, 5, and 10 μg/mg) for the selected receptor to determine the optimum concentration for promoting bone formation. Our data demonstrated that the mechanoactivation of integrins (CD49e) significantly upregulated the RUNX2 and LEF1 genes compared to other selected receptors. Furthermore, comparing the mechanoactivation of cells using MNPs conjugated with CD49e antibodies and aptamers revealed that MNP–aptamers significantly enhanced the upregulation of LEF1 genes. This suggests that aptamer-mediated mechanoactivation is a promising alternative to antibody-mediated activation. Finally, our results showed that the concentration of the aptamer loaded onto the MNPs strongly influenced the mechanoactivation of the cells. These findings provide valuable insights into the use of MNP platforms for bone regeneration and highlight the potential of aptamers in promoting signaling pathways related to bone formation. The novelty of our study lies in elucidating the unique advantages of aptamers in mediating mechanoactivation, presenting a promising avenue for advancing bone regenerative strategies.

## 1. Introduction

Bone regeneration and repair are complicated processes in the adult skeleton, with a high number of studies currently focusing on how to control and regulate these processes [1]. In bone regeneration, mesenchymal stromal bone-marrow-derived cells (MSCs) have the potential to differentiate into osteoblast cells and finally mature into osteocytes to remodel the bone [2]. MSCs have already illustrated considerable potential and therapeutic value in the regeneration of bone in several in vitro and in vivo models or repair [3,4]. However, for effective stem cell therapy, the consistent regulation of differentiation via transcription factors and signaling pathways is essential. Two key transcription factors, Runt-related transcription factor 2 (RUNX2) and Osterix/Sp7 (Osx), play an important role as key regulators in osteogenic differentiation [5,6]. These factors regulate several signaling pathways involved in osteogenic differentiation, including BMP, IGF, Akt, and Wnt [7,8]. Wnt signaling has been found to play a key role in osteogenic differentiation, with its natural age-associated reduction leading to bone repair dysfunction [8,9], and this pathway can potentially be manipulated through the activation of the Frizzled receptor on the cell membrane [10]. Recent studies demonstrated that Piezo1, as a mechanosensitive channel, can transduce mechanical forces into cellular signals and can play very essential roles in many biological mechanisms [11,12]. Sun et al. reported that Piezo1 can control the formation and mechanical-loading-dependent regeneration of the bone in murine models and can be a target to help curtail bone loss in osteoporosis [13]. Our previous research investigated the effect of the dynamic magnetic force activation of Piezo1 on osteogenesis [14].

Magnetic nanoparticle (MNP)-based platforms have been demonstrated to play roles in tissue engineering and regenerative medicine via the use of magnetic materials [15,16]. Properly stabilized magnetic nanoparticles display superparamagnetic properties and can be conjugated with receptor targeting biomolecules. When a remote dynamic field gradient is applied, the MNPs move in the changing gradient and apply a mechanical force onto the mechanoreceptors on the cell surface. We have previously demonstrated how this mechanoactivation can then lead to downstream signaling [17,18] and ultimately new bone formation [19,20]. This technique with progenitor cells from multiple sources has been shown to promote differentiation toward musculoskeletal cell types [19]. For instance, it has recently been shown that the osteogenic signaling pathways, such as calcium signaling, tyrosine phosphorylation, MAPK activation, Wnt signaling activation, AKT phosphorylation, PDGFR phosphorylation, and SMAD2/3 phosphorylation, can be modulated through the magnetic field and using MNP–peptides or antibodies targeted against receptors such as integrins, TREK1, PDGFR, Frizzled, Activin A, and EGFR [15]. We have already demonstrated in vitro and in vivo that integrins [21], Wnt receptors [22], and ion channels [14,19] can be activated using this technology, and this can be applied to improve bone formation in tissue engineering models.

However, these studies did not optimize the control of these signaling pathways, and it is not clear which receptor is most efficacious for initiating the downstream expression of RUNX2 and the upregulation of Wnt signaling for osteogenesis. Therefore, our first aim in this study was to use MNP platforms to compare the mechanoreceptors known to promote osteogenesis, e.g., Piezo1, Fzd1, Fzd2, and integrin alpha-5, and assess the degree of upregulation of the expression of the RUNX2 and Wnt signaling pathway for osteogenesis.

Furthermore, we assessed the potential of different types of targeting biomolecule/MNP complex to optimize the binding and activation. Aptamers are short nucleic acid sequences capable of specific, high-affinity molecular binding, which bind to proteins and receptors with a higher affinity than antibodies. Aptamers have several advantages, including a small size, a flexible structure, good biocompatibility, and low immunogenicity [23,24,25]. In this study, we compared agonists (antibody vs. aptamer) in terms of binding to cell surface receptors using the MNP platform as our second main aim. Finally, we optimized the concentration of the aptamer added to the MNPs (2.5, 5, or 10 μg/mg) for the selected receptor in order to determine the optimum aptamer concentration for promoting bone formation.

## 2. Materials and Methods

### 2.1. Cell Culture

Human MSCs (hMSCs) were obtained from a bone marrow aspirate (21-year-old healthy male, Lonza, Walkersville, MD, USA) and cultured to passage three in basal Dulbecco’s modified Eagle’s medium (DMEM) containing 10% fetal bovine serum (FBS), 1% L-glutamine (LG), and 1% penicillin–streptomycin (P/S). Media were replaced twice per week, and cells P1-4 were used in all experiments. Y201 MSCs cells [26] and Y201 TCF-LEF GFP reporter cells [27] were provided by Prof. Paul Genever (Biology, University of York) and cultured in DMEM including 10% FBS, 1% LG, and 1% P/S. Osteogenic medium (OM) was prepared via the addition of 0.2 mM ascorbic acid (Sigma, Gillingham, UK), 10 mM sodium β-glycerophosphate (Sigma), and 0.1 μM dexamethasone (Sigma) to the growth media. For the positive control in Y201 TCF-LEF GFP reporter cells, Wnt-conditioned medium was prepared by collecting basal media cultured with Wnt3a-overexpressing L-M (TK-) cells (ATCC, Teddington, UK); the Wnt-conditioned medium was diluted 1:5 with fresh basal media before use [22].

### 2.2. MNPs: Ligand Labeling

**Figure 1:**
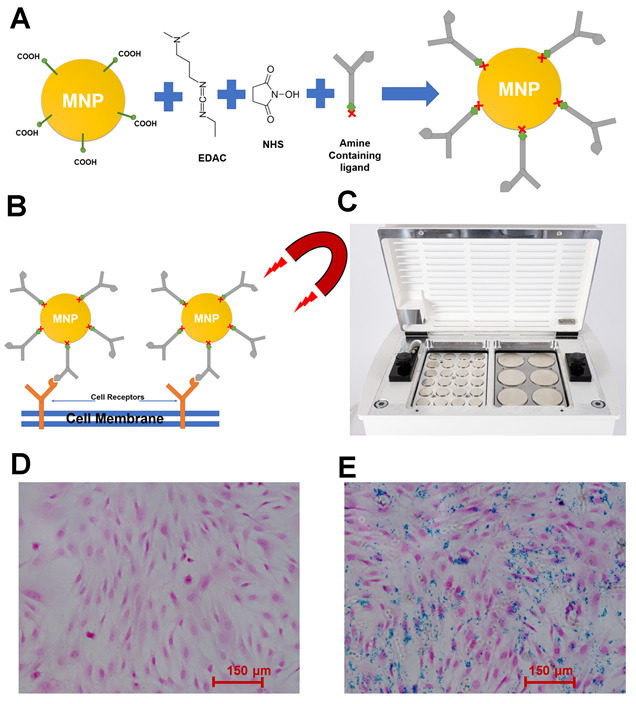
(**A**) Schematic image of functionalizing MNPs with ligands using EDAC and NHS; (**B**) schematic image of labeling cells with MNP–ligand under magnetic field; (**C**) image of the magnetic force bioreactor used in magnetic stimulation experiments. Culture plates were situated on the plate holder above the magnetic array, which oscillated vertically beneath the culture plates. The movement parameters for the array were controlled with a computer. (**D**) Nuclear Fast Red (NFR) and Prussian Blue (PB) staining of hMSCs without labeling with MNP–ligand. (**E**). Nuclear Fast Red (NFR) and Prussian Blue (PB) staining of hMSC labeled with MN–ligand.

Nanomag superparamagnetic nanoparticles (dextran-coated, 250 nm in diameter; magnetization: 43 Am^2^/kg iron, saturation magnetization: >63 Am^2^/kg iron; nanomag^®^-D; (Micromod, Rostock, Germany) were surface-activated according to a previously published protocol (Figure 1A) [19]. Briefly, the MNPs were washed in sterile 1-ethyl-3-(3-dimethylaminopropyl)- carbodiimide hydrochloride and N-hydroxysuccinimide in 0.5 M (2-(N-morpholino) ethanesulfonic acid] MES buffer, adjusted to pH 6.3 at room temperature for 1 h, recovered via magnetic separation, and washed in 0.1 M MES buffer. Then, 1 mg of MNPs was conjugated overnight to either 10 µg of Piezo1 antibody (Proteintech, Manchester, UK), Fzd1 antibody (ABclonal, Port Talbot, UK), Fzd2 antibody (Thermo Fisher, Waltham, MA, USA), and CD49e antibody (integrin α5, Thermo Fisher), or 10 µg of Fzd1 aptamer (Aptamer Sciences, Pohang, Republic of Korea), and CD49e/CD29 aptamer (integrin α5β1, Aptamer Sciences). Moreover, 1 mg of MNPs was conjugated with 2.5, 5, 10, or 20 µg of CD49e/CD29 aptamer to optimize the concentration of integrin aptamer. All aptamers were amine-tagged and were bought from Aptamer Sciences. The size and surface charge of MNPs with or without ligand conjugation was measured using a ZetaSizer (Malvern Panalytical, Malvern, UK) at 25 °C when dispersed in distilled H_2_O.

### 2.3. Loading Efficiency

In this experiment, we utilized a Qubit™ ssDNA Assay Kit (Invitrogen, Waltham, MA, USA). This kit uses an ultrasensitive fluorescent nucleic acid stain to allow quantification, via fluorescence intensity measurement in a plate reader, of the amount of single-stranded DNA (ssDNA) in solution. The blank aptamers were dissolved in the TE buffer supplied by the kit in a series of dilutions to prepare a standard curve. All samples were treated following the manufacturer’s instructions. Then, we measured the fluorescent intensity of the samples (excitation wavelength: 480 nm; emission wavelength: 530 nm) using a TECAN Spark^®^ microplate reader (TECAN, Reading, UK). After the preparation of the standard curve, we measured the florescence intensity of MNP–aptamers using the same kit and calculated the loading efficiency using the measured amount of aptamer divided by the total aptamer added.

### 2.4. Cell MNP Labeling

The cells were labeled with MNP following on a previously published protocol (Figure 1B) [14,28]. Briefly, medium was removed, and cells were washed with PBS. All cells were cultured in reduced serum basal media (1% FBS) for 3 h. Particles were added to appropriate groups at approximately 3 μg MNP/cm^2^ of culture surface area and incubated for a further 1.5 h with intermittent agitation. Medium was aspirated and cells were washed with PBS to remove unbound particles before addition of fresh reduced serum basal media (1% FBS) for the experiments. The control samples were cells without labeling with MNPs. In some experiments, soluble aptamers (the concentration of aptamer in the basal media was same as the concentration of aptamer in MNP-10 aptamer) were also used as another control. For the positive control, OM medium or Wnt 3A-conditioned medium collected from Wnt 3 A-overexpressing L-M(TK-) cells (ATCC) was used. To verfiy the labeling of cells with MNP, cells were fixed with 4% paraformaldehyde for 10 min, and iron was detected via Prussian Blue staining (2% potassium ferrocyanide in 1% HCl) followed by counterstain with Nuclear Fast Red (ScyTek Lab., Logan, UT, USA) based on the manufacturer’s instructions.

### 2.5. Magnetic Field Gradients

Magnetically stimulated groups were treated with ≥25 mT magnetic fields provided by arrays of NdFeB magnets in 1 h sessions at 1 Hz using a vertically oscillating magnetic force bioreactor (Figure 1C), (MICA Biosystems, Birmingham, UK). The bioreactor was housed in a cell culture incubator maintained at 37 °C, 5% CO_2_.

### 2.6. qRT-PCR

After labeling the cells, the cells were magnetically stimulated for 1 h. Then, 24 h after stimulation, the RNA was extracted using an RNAeasy extraction kit (Qiagen, Manchester, UK) according to the manufacturer’s instructions. Reverse transcription was performed on 700 ng of RNA using a High Capacity cDNA Reverse Transcription Kit (Applied Biosystems, Warrington, UK) to synthesize cDNA. The obtained cDNA was used to conduct qRT-PCR on an AriaMx Real-Time qPCR system using SYBR Green Master Mix (Applied Biosystems, Thermofisher Scientific, Waltham, MA, USA) and primers (Qiagen) following the manufacturer’s protocol. qRT-PCR was performed to analyze the expression of Runt-related transcription factor 2 (RUNX2) and lymphoid enhancer binding factor 1 (LEF1). The CT values were normalized to the glyceraldehyde 3-phosphate dehydrogenase (GAPDH) housekeeping gene, and fold induction was calculated using the comparative ΔCT method.

### 2.7. Flow Cytometry

After labeling Y201 TCF-LEF GFP reporter cells with MNP, they were magnetically stimulated for 1 h; 24 h later, they were stimulated for 1 h; and after 24 h (48 h from the first stimulation), they were detached, centrifuged, fixed, and resuspended in PBS (including 1% BSA, 0.1% NaN_3_ sodium azide) to analyze and quantify the GFP expression via flow cytometry. A CytoFlex flow cytometer (Beckman Coulter, High Wycombe, UK) equipped with a laser (488 nm) capable of GFP excitation was utilized for quantification of GFP expression. The threshold was set using a green fluorescence detector to just above the majority of nonfluorescent cells in the control group. CytExpert software (Version 2.4.0.28) was used to analyze the flow cytometry data and calculate the percentage of positive GFP cells. Furthermore, all cells (hMSCs, Y201, and Y201 reporter) were characterized for expression of surface markers (CD73 and CD49e) before magnetic stimulation using CD73 antibody (BD Bioscience, Wokingham, UK) and CD49e antibody (Thermo Fisher) and the relevant laser filter in CytoFlex.

### 2.8. ALP Activity

After 3 and 7 days of magnetic stimulation (1 h per day), the ALP enzymatic activity was quantitatively measured using aSensolyte ALP assay kit (Cambridge Bioscience, Cambridge, UK). Briefly, the samples were treated with lysis buffer for 10 min. Then, we gently scraped the cells and collected the cell suspension in a microcentrifuge tube. We centrifuged the cell suspension at 2500× *g* for 10 min at 4 °C and then added 100 µL of p-nitrophenyl phosphate (pNPP) solution into each well. Then, we gently shook the plate for 30 s. After 1 h, the absorbance was measured at 405 nm using the microplate reader. The ALP concentration was measured and calculated using the standard curve against the known protein concentrations. Moreover, the total protein of each sample was quantified using a BCA assay kit (Thermo Scientific, Waltham, MA, USA). Finally, the amount of ALP normalized to the amount of total protein per sample.

### 2.9. Western Blotting

Cells were lysed with RIPA buffer (Thermo Scientific) containing a protease and phosphatase inhibitor mix (Sigma). The cell lysate was clarified, and the total protein of each sample was quantified using a BCA assay kit (Thermo Scientific). For PAGE, 10 μg of protein was mixed with LDS sample buffer and β-mercaptoethanol (Invitrogen). Samples were then heated for 5 min at 95 °C and briefly centrifuged before being loaded onto a Tris-glycine 4–20% gel in Tris-Gly running buffer (Nusep-Generon, Slough, UK). Proteins were transferred to a nitrocellulose membrane (Novex, ThermoFisher), which was then blocked with 5% milk powder (Bio-rad, Watford, UK) or 5% BSA in TBST buffer (Sigma) based on the antibody protocol. The membrane was then incubated with anti-RUNX2 (Bio-techne, Abingdon, UK), anti-osteopontin (Abcam, Cambridge, UK), and anti-GAPDH (Abcam) overnight at 4 °C under constant mixing. The membrane was washed with TBST 3× before incubation with anti-rabbit-HRP, anti-goat-HRP, or anti-mouse-HRP (1:1000) (Abcam) for 1 h at room temperature. The membrane was washed 5× in TBST, and chemiluminescence was developed using a SuperSignal WestPico PLUS chemiluminescent kit (Thermo Scientific) followed by image capture using an iBright 1500 imaging system. The image was analyzed using ImageJ, and the data obtained from RUNX2 and osteopontin was normalized to the GAPDH.

### 2.10. Alizarin Red Staining

After 3 weeks of magnetic stimulation of Y201 cells (stimulation for 1 h per day, media changed every 2–3 days), spent media were removed, and cells were washed three times with calcium- and magnesium-free PBS. Cells were fixed with 4% paraformaldehyde for 10 min at room temperature and washed a further three times with distilled H_2_O. Alizarin Red staining solution (40 mM, pH 4.1, sterile filtered) (Sigma) was added to the cells, which were incubated for 10 min with gentle agitation. The staining solution was then removed, and cells were washed three times with dH_2_O. Cells were immediately imaged using a microscope. To extract the stain and quantify the calcification, 10% (*v*/*v*) acetic acid was added to the wells. Cells were incubated for 30 min with gentle agitation. Cells were scraped, transferred to microcentrifuge tubes, and vortexed for 30 s. Samples were then heated to 85 °C for 10 min and subsequently cooled on ice prior to centrifugation at 10,000× *g* for 15 min. Supernatants were then neutralized with 10% ammonium hydroxide before loading in triplicate into an opaque-walled 96-well plate; absorbance was measured at 405 nm using a plate reader.

### 2.11. Statistical Analysis

All data are presented as means ± standard error of the mean (SEM). Statistical significance at the 95% confidence level was determined using 1-way ANOVA with post hoc Tukey’s tests, and statistically significant differences are marked with * for *p* < 0.05, ** for *p* < 0.01, *** for *p* < 0.001, and **** for *p* < 0.0001 in the figures.

## 3. Results and Discussion

### 3.1. Cell Tagging with MNP

Dextran–MNPs was functionalized with anti- Piezo1, Fzd1, Fzd2, and integrin alpha-5 antibodies, followed by binding to MSCs, as illustrated in Figure 1A,B. Prussian Blue staining confirmed the attachment to the cell membranes, with nanoparticles adherent to cells indicated in blue. Cells without MNPs did not stain with Prussian Blue, indicating that no iron0based particles were present (Figure 1D,E). Figure 1C shows the MICA instrumentation used to apply dynamic changes in the field gradient onto multiwell plates in vitro.

The antibody-functionalized MNPs were subjected to characterization via dynamic light scattering (DLS) and zeta potential analysis to evaluate the alterations in the particle size and surface charge after antibody coating (Table 1). The outcomes indicated a distinct change in surface potential following antibody conjugation, indicative of the modifications of the surface charge resulting from the deposition of antibodies onto the MNP surface. However, a less apparent shift in size was observed after antibody conjugation. These findings broadly align with those of our prior investigations [17].

### 3.2. Receptor Selection for Osteogenesis

**Figure 2:**
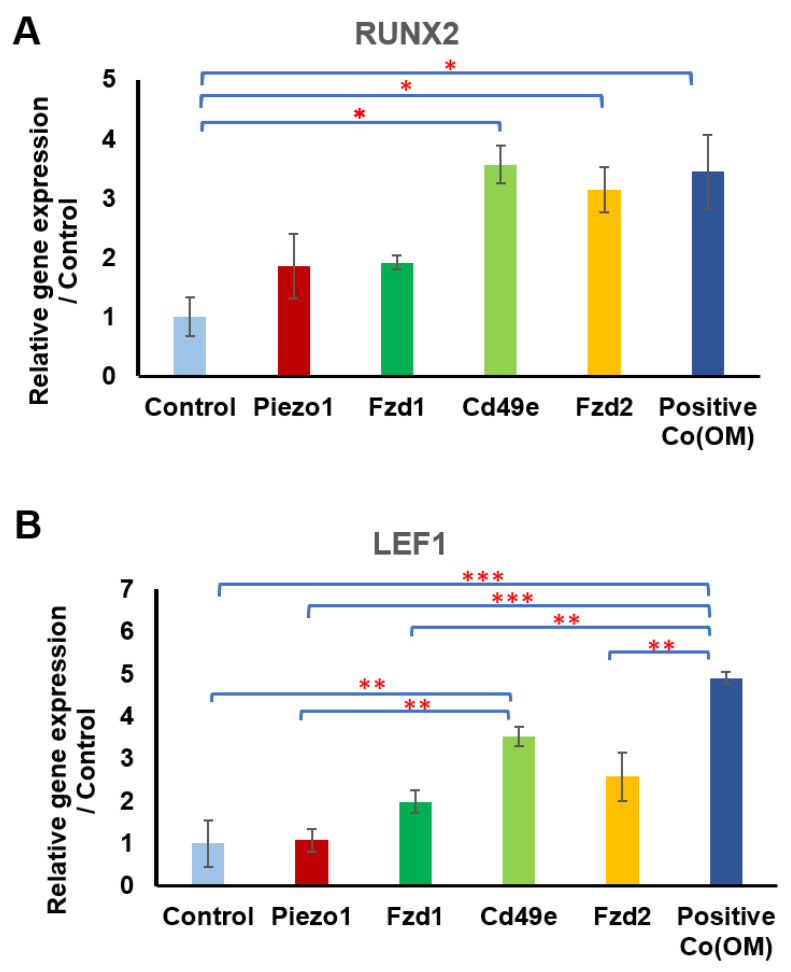
Among targeted different receptors (Piezo1, Fzd1, CD49e, and Fzd2), magnetic stimulation of hMSCs by MNP-CD49e antibody significantly upregulated expression of RUNX2 and LEF1 genes. (**A**) Gene expression analysis of magnetically stimulated hMSCs with different MNP–ligands of RUNX2 after 24 h of stimulation in reduced serum basal media (1% FBS) compared to control (cells without MNP–ligands) and positive control (osteogenic media). (**B**) Gene expression analysis of magnetically stimulated hMSCs for different MNP–ligands for LEF1 after 24 h of stimulation in reduced serum basal media (1% FBS) compared to control (cells without MN–ligands) and positive control (osteogenic media). Data presented as mean ± SEM (*n* = 3), and statistically significant differences are marked with * for *p* < 0.05, ** for *p* < 0.01, and *** for *p* < 0.001.

Gene expression analysis of RUNX2 and LEF1 in mechanoactivated hMSCs tagged with different conjugated MNPs was performed to monitor the early markers of osteogenesis and Wnt signaling, respectively (Figure 2). The analysis of the data from the mechanoactivation of the CD49e (integrin α5) samples indicated a significant upregulation of genes RUNX2 (3.56-fold) and LEF1 (3.52-fold) relative to the control. The mechanoactivation of other receptors including Fzd2, Fzd1, and Piezo1 showed the upregulation of RUNX2 by 3.13-, 1.91-, and 1.85-fold, respectively; and the upregulation of LEF1 with 2.57-, 1.97-, and 1.07- fold, respectively, compared to the control.

Fzd1 and Fzd2 receptors can both interact with the *WNT3A* ligand and play very important roles in the activation of Wnt signaling pathways and osteogenesis [29]. hMSCs express Fzd receptors, which enable them to bind to specific Wnt ligands [22,30]. We previously studied the dynamic magnetic force activation of Fzd receptors using a synthetic peptide (UM206, a ligand for the Fzd1 and Fzd2 receptors) and Fzd2 antibody conjugated to the MNPs [22,28]. Stimulation of the Fzd2 receptor significantly increased the expression of RUNX2 compared to the control. Although the family of Wnt signaling and Wnt receptors play key roles in bone biology and remodeling [8,31], it has been reported that among the Wnt receptor family, Fzd2 can play a crucial role in skeletal development [32]. Figure 2A shows the significant upregulation of RUNX2 in response to the dynamic activation of the Fzd2 receptor compared to Fzd1 activation. Interestingly, however, a small but not statistically significant elevation was observed in the LEF upregulation in response to Fzd1 and Fz2 receptor activation.

The fibronectin receptor CD49e (integrin α5) is involved in stem cell differentiation [33,34]. Integrin stimulation using RGD-MNP can promote bone markers and bone formation in osteoblasts and MSCs [21]. CD49e plays a role in the biological responses to mechanical stimuli in MSCs under hydrostatic pressure [35]. In addition, it has been shown that integrin α5β1 is responsive to internal forces and fluid shear force [36]. Further studies have defined CD49e alone or with CD29 (integrin α5β1) as mechanosensitive receptors [37,38]. CD49e also plays a role in the osteogenic differentiation of bone-marrow-derived mesenchymal progenitors and in bone regeneration [39,40]. It can be upregulated during progenitor cell commitment and osteogenic differentiation. In fact, there is a significant increase in the expression of receptor CD49e during differentiation of MSCs into osteoblasts [40]. Our results agree with these findings in that the mechanoactivation of the CD49e receptor through dynamic magnetic force activation can significantly upregulate osteogenesis and Wnt signaling compared to other targets (Piezo1, Fzd1, and Fzd2), to levels similar to those observed for the positive control. Our work also demonstrates the signaling crosstalk between integrin and Wnt signaling. Other studies showed that Wnt/β-catenin signaling can be activated through the integrin/focal adhesion kinase (FAK) pathway in response to matrix stiffness [41]. Furthermore, our results show how the upregulation of the expression of RUNX2 by the magnetic stimulation of a target (CD49e) in basal media (including low concentration serum) was comparable to the upregulation of RUNX2 induced by the positive control (osteogenic media). In this study, we investigated the feasibility of exploiting the stimulation of a cell receptor to develop remote mechnoactivation platforms for driving the osteogenic differentiation of hMSCs without the use of any metabolic, osteogenic, or growth factors. Our data highlight the importance of the mechanosensitive CD49e target, which has a key role in osteogenesis, so was selected for further studies.

### 3.3. Comparison between Aptamer and Antibody

**Figure 3:**
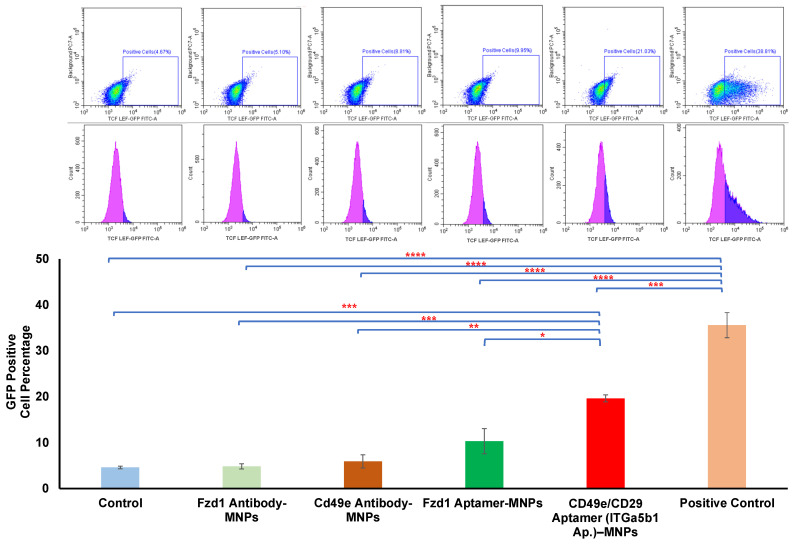
Comparison of TCF/LEF GFP reporter in Y201 cells magnetically stimulated with MNP–antibodies (MNP-Fzd1 antibody and MNP-CD49e antibody) and MNP–aptamers (MNP-Fzd1 aptamer and MNP CD49e/CD29 aptamer). MNP-CD49e/CD29 aptamers significantly activated the Wnt TCF/LEF GFP reporter compared to MNP–antibodies in Y201 cells. Data are presented as mean ± SEM (*n* = 3), and statistically significant differences are marked with * for *p* < 0.05, ** for *p* < 0.01, *** for *p* < 0.001, and **** for *p* < 0.0001.

A key factor in the dynamic magnetic activation approach is the tagging of specific regions of the receptor, which may be a mechanically responsive region. Different strategies can be used for targeting different regions of the receptor protein using aptamer and antibody approaches. Aptamers are thermally and chemically more stable than antibodies, and they are nonimmunogenic [23,42,43]. They can also specifically target receptors; importantly, they can be chemically modified without losing their functionality [42,43]. To explore the potential variation in aptamer binding and antibody strategies, antibody and aptamers for Fzd1 and CD49e receptors were conjugated to MNPs, and their signaling efficacy was investigated using Y201 TCF/LEF GFP reporter cells. The aptamers used were raised against combined CD49e/CD29 receptors. The analysis of the data obtained from flow cytometry from the reporter cells demonstrated that the mechanoactivation of the cells by MNPs conjugated with the CD49e/CD29 aptamer resulted in the significant upregulation of GFP expression in TCF/LEF reporter cells, an initial factor indicating the downstream activation of the Wnt signaling pathway (Figure 3). Signaling activation using aptamer-conjugated MNPs was significantly higher than that of antibodies raised against CD49e. Previous studies demonstrated the existence of specific sites recognized by CD49e/CD29 integrins, which can be activated in response to mechanical forces [37]. It has also been shown that the heterodimer of CD49e integrin and CD29 integrin, working together rather than individually, plays a crucial role in fibronectin binding and the subsequent activation of downstream signaling pathways [35,36,37,44]. Therefore, targeting the dimer of CD49e/CD29 integrin using an aptamer may enhance the activation of receptors compared to the activation with antibodies to CD49e alone in response to a dynamic magnetic force.

The number of papers that report o the application of aptamers in the different fields of biomedical research has substantially risen. Research has focused on developing aptamer-based technologies for diagnostics as well as targeted therapy for targeting drug delivery using nanoparticles modified using aptamers [42,45]. For the first time in this study, we investigated the application of an aptamer as a conjugate on MNPs for activating mechanoreceptors for regenerative medicine. Our data demonstrate the potential application of receptor targeting with aptamers for the mechanostimulation of clustered integrin targets.

**Figure 4:**
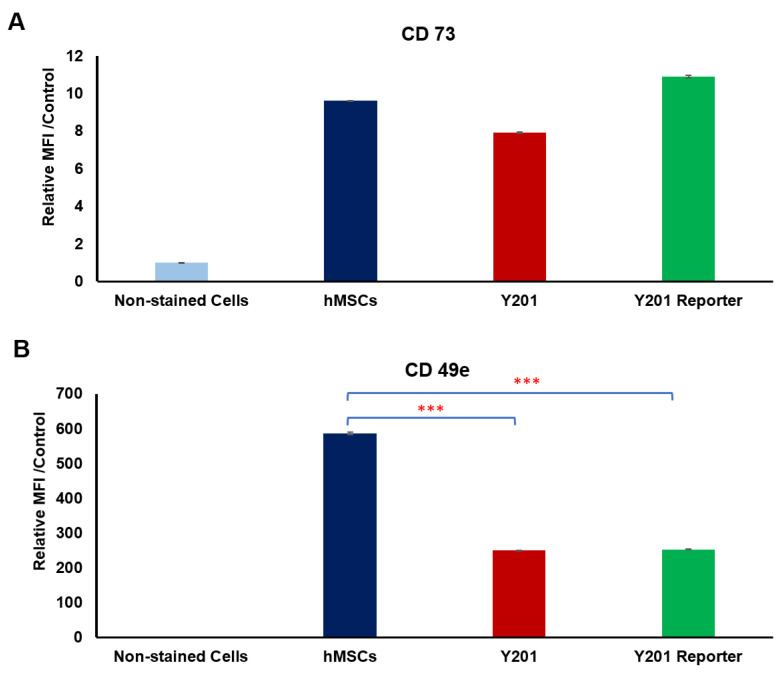
(**A**) Expression of CD73 in different cells (hMSCs, Y201, and Y201 reporter cells). (**B**) Expression of CD49e in different cells (hMSCs, Y201, and Y201 reporter). There was a significant reduction in the expression of CD49e in Y201 and Y201 reporter cells compared to hMSCs. Data are presented as mean ± SEM, and statistically significant differences are marked with *** for *p* < 0.001.

We have analyzed the expression of two CD markers (CD73, positive marker for MSCs and Y201 cells [26,46,47], and CD49e) across osteogenic progenitor primary cell and cell lines (hMSCs, Y201, and Y201 TCF/LEF reporter cells) to confirm the mesenchymal state of the cells (Figure 4). The levels of the MSC marker CD73 were similar across the cell types examined (Figure 4A), but, interestingly, the expression of CD49e (α5-integrin subunit) was lower in Y201 and Y201 reporter cells than in hMSCs. hMSCs and Y201 cells showed a similar level of the expression of CD73 as a mesenchymal stem cell marker. James et al. [26] analyzed the signaling pathways between Y201 cells and their parent (hMSCs), and they found some of the signaling pathways including Wnt signaling can be impacted by cloning of Y201 cells.

### 3.4. Optimization of Aptamer Concentration

The aptamer-functionalized MNPs were characterized using DLS and zeta potential to assess the changes in particle size and surface charge after aptamer coating (Table 2). The results demonstrated no noticeable shift in the surface potential and a minimal change in size after aptamer conjugation. Interestingly, varying the concentration of the aptamer did not induce significant alterations in surface potential, suggesting a similarity between the surface charge of the nanoparticles and the charges of the aptamers.

**Figure 5:**
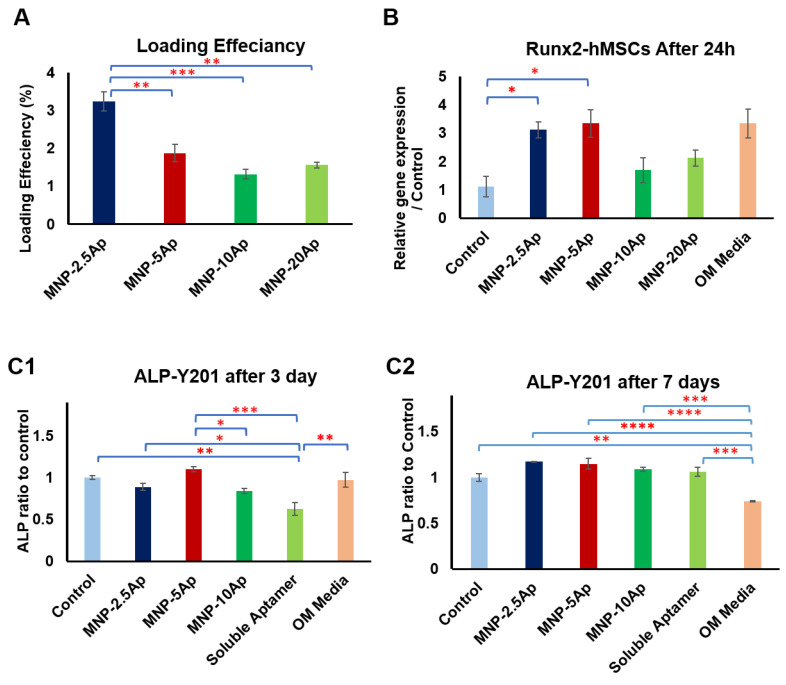
For different concentrations of CD49e/CD29 aptamers (2.5, 5, 10, and 20 µg), magnetic stimulation of cells using MNP-2.5 and -5 aptamers significantly upregulated expression of RUNX2 and ALP. (**A**) Loading efficiency of aptamer in four different groups of prepared MNP–aptamers. (**B**) RUNX2 gene expression analysis of magnetically stimulated hMSCs with different concentrations of aptamers loaded on MNPs after 24 h of stimulation in reduced serum basal media (1% FBS) compared to control (cells without MNP–aptamer) and positive control (osteogenic media). (**C1**) and (**C2**) ALP activity of magnetically stimulated Y201 cells with different concentrations of aptamers loaded onto MNPs after 3 days and 7 days of stimulation in reduced serum basal media (1% FBS) compared to control (cells without MNP–aptamer), soluble aptamer, and positive control (osteogenic media). Data are presented as mean ± SEM (*n* = 3), and statistically significant differences are marked with * for *p* < 0.05, ** for *p* < 0.01, *** for *p* < 0.001, and **** for *p* < 0.0001.

The loading efficiency of the aptamers onto MNPs through EDAC functionalization ranged from 1.3% to 3.2% (Figure 5A). The data revealed that the loading efficiency of aptamer was 3.2% ± 0.2 in MNP-2.5 Ap, 1.9% ± 0.2 in MNP-5 Ap, 1.3% ± 0.2 in MNP-10 Ap, and 1.5% ± 0.1 in MNP-20 Ap. Taking into consideration the number of MNPs per milliliter (4.9 × 10^10^) and the molecular weight of the aptamer (2.4 × 10^4^ g/mol), the loading efficiency was estimated as approximately 3.9 × 10^1^, 4.6 × 10^1^, 6.4 × 10^1^, and 1.5 × 10^2^ molecules of aptamer on the surface of each MNP in the MNP-2.5 Ap, MNP-5 Ap, MNP-10 Ap, and MNP-20 Ap samples, respectively. However, the loading efficiency decreased as the initial amount of aptamer added to the MNPs increased, potentially due to the saturation of the active sites during functionalization. The total amount of aptamers attached to the surface of the MNPs was increased by increasing the addition of aptamers to the MNPs. When normalizing the total amount of aptamers in each sample group to the total amount of the aptamer in MNP-2.5 Ap, the relative amount of rhe aptamer was 1 for MNP-2.5 Ap, 1.16 for MNP-5 Ap, 1.63 for MNP-10 Ap, and 3.86 for MNP-20 Ap. This demonstrated that increasing the addition of aptamer from 2.5 to 20 μg (an eight-fold increase) resulted in a practical increase of 3.86 times after the reaction and washing steps.

The concentration of binding sites on the surface of the MNPs was explored using the dose–response of CD49e/CD29 aptamer (2.5, 5, 10, and 20 µg) and analysed by gene expression of RUNX2 (Figure 5B). The analysis of data showed that dynamic magnetic force stimulation of cells tagged with MNPs conjugated with 2.5 and 5 µg of aptamer in low serum basal growth medium significantly upregulated the gene expression for RUNX2 relative to the control. Interestingly, their response was very similar to that of RUNX2 expression induced using osteogenic media. The response of the upregulation of RUNX2 was reduced by using MNP-10 Ap and MNP-20 Ap.

In addition, we defined the differences in the downstream osteogenic response between the addition of soluble aptamer versus MNP-bound aptamers in varying concentrations. Following the application of a dynamic magnetic force to Y201 cells tagged with MNPs conjugated with different concentrations of CD49e/CD29 aptamer (2.5, 5, and 10 µg), we evaluated the ALP activity after 3 days and 7 days (Figure 5C1,C2). The data showed that the ALP activity of the cells tagged with MNP-2.5 Ap and MNP-5 Ap was significantly higher than the activity of those tagged with MNP-10 Ap or soluble aptamers in the media. In addition, the ALP activity of the cells in osteogenic media did not increase compared to that of the cells in basal media at day 3 and day 7; this could have been due to the early timing (day 3 and day 7) of the investigation into ALP activity, as other studies also demonstrated that ALP is not detectable in the early stage of MSC differentiation and in immature osteoprogenitors [26,48,49].

**Figure 6:**
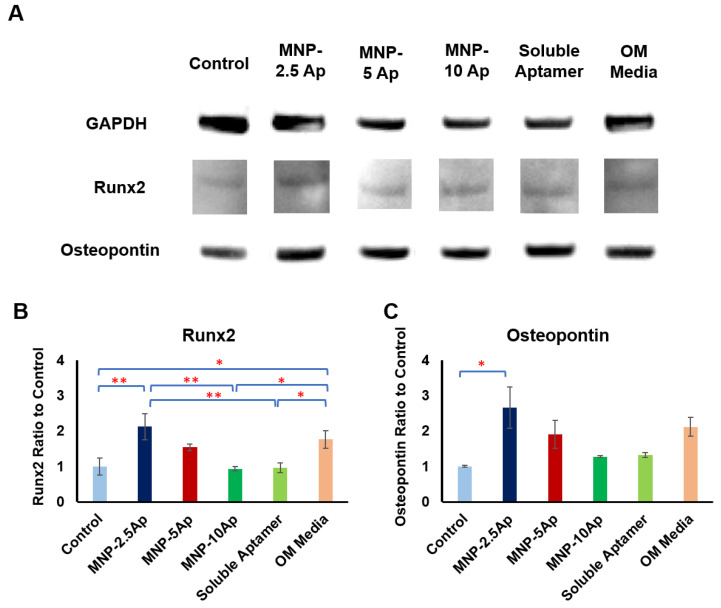
For different concentrations of CD49e/CD29 aptamers (2.5, 5, and 10 µg), magnetic stimulation of Y201 cells by MNP-2.5 aptamers significantly upregulated expression of RUNX2 and osteopointin after 7 days. (**A**) Western blot images of magnetically stimulated hMSCs for different concentrations of aptamers loaded on MNP for GAPDH, RUNX2, and osteopontin after 7 days of stimulation in reduced serum basal media (1% FBS) compared to control (cells without MNP–aptamer), soluble aptamer, and positive control (osteogenic media). (**B**) Quantified data obtained from image analysis of Western blotting for expression of RUNX2 at the protein level in Y201 cells on day 7. (**C**) Quantified data obtained from image analysis of Western blotting for expression of osteopontin protein level in Y201 cells on day 7. Data are presented as mean ± SEM (*n* = 3), and statistically significant differences are marked with * for *p* < 0.05, and ** for *p* < 0.01.

Moreover, we evaluated the expression of RUNX2 and osteopontin at the protein level in these cells on day 7 using Western blot (Figure 6). As shown in Figure 6, MNP-2.5 Ap significantly promoted the expression of RUNX2 and osteopontin at 7 days compared to the controls. Despite the fact we were not able to see a significant difference between MNP-2.5 Ap and MNP-5 Ap in the PCR study and ALP study, the Western blot data demonstrated that MNP-2.5 Ap had a stronger ability to upregulate osteogenic marker expression than MNP-5 Ap after 7 days of differentiation.

**Figure 7:**
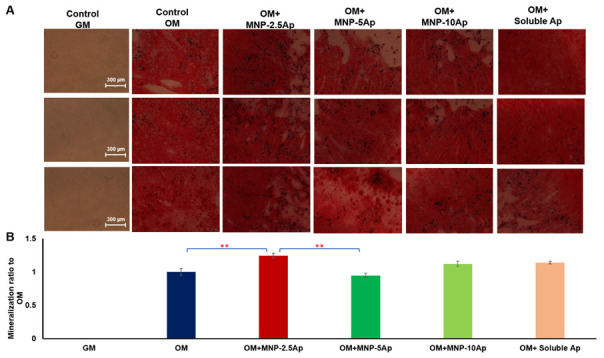
For different concentrations of CD49e/CD29 aptamers (2.5, 5, and 10 µg), magnetic stimulation of Y201 cells by MNP-2.5 aptamers significantly enhanced mineralization after 3 weeks. (**A**) Alizarin staining images of magnetically stimulated Y201 cells with different concentrations of aptamers loaded on MNPs after 3 weeks of stimulation in osteogenic media. (**B**) Quantified mineralization results following elution of Alizarin Red S and spectrophotometric measurement. Data are presented as mean ± SEM (*n* = 3), and statistically significant differences are marked with ** for *p* < 0.01.

Furthermore, Alizarin Red staining was conducted after 3 weeks in Y201 cells, which were labeled with different concentration of CD49e/CD29 aptamer (2.5, 5, or 10 µg) added to the MNPs; this was performed in order to evaluate the matrix mineralization and to optimize the concentration of the aptamers on the MNP surface (Figure 7A,B).

Alizarin Red staining and quantification analyses illustrated a significant increase (*p* < 0.01) in mineralization when Y201 cells were loaded with MNP-2.5 Ap and magnetically stimulated over 3 weeks of induction. Microscopy demonstrated an improvement in calcification around the cells in the MNP-2.5 Ap samples (Figure 7A). As can be seen, the intensity for Alizarin Red S staining progressively enhanced with the mechanoactivation of cells with MNP-2.5 aptamer in osteogenic media. On the contrary, lower calcification or no calcification was observed for the other groups of samples. The Alizarin Red staining data were consistent with the Western blot data, which showed adding a lower concentration of aptamer (2.5 µg) to the MNP was more functional in activating the cells for long period for osteogenic differentiation and orthopedic applications.

A ligand is able to activate receptors through different processes, including conformational change of the receptor, small- or large-scale clustering of multiple receptors, or slowing receptor diffusion across cell membranes [50]. The concentration and density of ligand play very important roles in the activation of signaling pathways and, consequently, cell fate [17,50,51,52]. Kilian et al. demonstrated that the molecular characteristics of the adhesion ligands, including the density, influence the cytoskeleton of the cell and the differentiation pathways of MSCs [52]. Mann et al. [53] and Neff et al. [54] demonstrated that a low concentration of cell adhesion ligand helps cell migration and proliferation, while a higher concentration impedes cell migration and proliferation, and cells growing on the surfaces with a greater ligand density produce less matrix [53]. Other studies have shown that the optimal activation of cells through ligand binding occurs at an intermediate level of ligand density [50,55]. Wulfing et al. [50] explained how less ligand can be more effective, and they reported that when antibodies are used in excess over their antigen target, the chance of receptor clustering is reduced dramatically. In fact, with an excess of high-affinity antibodies, most receptors are stably bound to a single antibody arm, and there are few free receptors available for binding to the second arm of a receptor-bound antibody to support receptor clustering. These results reflect the fact that cells require an optimal concentration of aptamer on the surface of MNPs for cell activation. Furthermore, the steric effects in ligand–receptor binding arise from the size, shape, and arrangement of molecules, impacting their interaction [56]. Steric hindrance occurs when bulky groups obstruct optimal binding [56]. Ligand concentration and distribution play a vital role, as steric effects and hindrance determine ligand–receptor activation. Therefore, the availability of ligands for binding, in terms of concentration and distribution, is crucial for successful receptor activation.

The mechanisms of mechanostimulation through MNP–antibody was investigated [57]. Studies have demonstrated how mechanical stimulation can induce functional conformation transitions in mechanoresponsive proteins, which modify their binding properties and enzymatic functions [58,59]. Recent studies proposed that a conformational change in the transmembrane domains receptor might be part of the receptor activation mechanism [17]. It was also shown that mechanical stimuli can modulate endocytosis [17]. Furthermore, recent work from our group showed that the dynamic magnetic force activation of Fzd2 receptors in hMSCs by a peptide–MNP may induce a degree of receptor clustering at the cell membrane [22]. It is possible that there is a surface concentration of the ligand that is optimal for attachment to receptors, potentially inducing both clustering and activation of downstream signaling. Therefore, the low concentration of aptamer loaded on MNPs (MNP-2.5 Ap) used in these experiments could have acted as a complex facilitator to facilitate receptor clustering and the interaction of multiple components of the integrin pathway for osteogenesis.

In addition to the observed effects of the concentration of aptamers on MNPs, our data reveal a distinct advantage in the mechanoactivation of aptamer-conjugated MNPs compared to the impact achieved using soluble aptamers alone. The unique characteristics of the aptamer–MNPs, including their binding, localized delivery, and mechanoactiviation, likely contribute to a more pronounced cellular response compared to the use of soluble aptamers in isolation. The mechanical forces applied through the magnetic field, coupled with the receptor-specific binding of aptamers, create a synergistic effect that is not comparable to the outcomes achievable with soluble aptamers alone. The use of this aptamer approach enables the selection of aptamers during screening that are biologically inactive and yet still capable of binding and activating through the mechanical induction of the receptors. This enhanced mechanoactivation offers a promising avenue for applications in bone tissue engineering and osteogenesis.

## 4. Conclusions

In this study, we examined the role of the mechanoactivation of different receptors linked to integrin and Wnt signaling in the osteogenic differentiation of MSCs and probed osteogenic gene and protein expressions. The data demonstrated that the mechanoactivation of integrins (CD49e) significantly enhances the upregulation of the RUNX2 and LEF1 genes compared to that of the other receptors, so were selected for further investigations. Moreover, we compared the mechanoactivation of cells via MNPs conjugated with CD49e antibodies and aptamers, and the data demonstrated that the mechanoactivation of cells by MNP–aptamers significantly enhanced the upregulation of LEF1 and RUNX2 genes and led to increased osteogenic marker expression and matrix mineralization. This suggests that aptamer-mediated mechanoactivation of signaling is a viable alternative to antibody-mediated activation. Finally, the results illustrated that the mechanoactivation of the cells was strongly influenced by the concentration of aptamer loaded on the MNPs. Taken together, our results suggest that the magnetic stimulation of cells with MNP conjugated with a relatively low dose of aptamer (2.5 µg of aptamer-conjugated MNPs) is a promising approach for bone tissue engineering and osteogenesis.

Our work introduces the superior efficacy of aptamer-mediated mechanoactivation in promoting osteogenesis, offering a unique approach for bone tissue engineering. This approach demonstrates how the effects of the mechanoactivation of aptamer–MNPs exceed those of the use of soluble aptamers alone. Future research should explore a broader spectrum of concentrations and potentially unveil more nuanced responses. In addition, while this study provides valuable insights, further in vivo experiments and clinical validations are essential to translate these findings into practical applications. The intricate interplay of multiple signaling pathways involved in osteogenesis necessitates comprehensive exploration, opening avenues for future investigations to unravel more complexities in bone regeneration processes.

## Figures and Tables

**Table 1:** Size and surface potential of MNPs with or without ligand conjugation.

	MNP-Uncoated	MNP-Piezo1	MNP-Fzd1	MNP-Cd49e	MNP-Fzd2
Size (nm)	263.6 ± 17.6	253.5 ± 23.17	273.7 ± 28.6	265.1 ± 15.1	270.8 ± 27.4
Zeta (mV)	−15.4 ± 0.6	−11.1 ± 0.4	−11.5 ± 0.6	−12.3 ± 0.1	−11.8 ± 0.8

**Table 2:** Size and surface potential of MNPs with aptamers conjugation.

	MNP-Uncoated	MNP-2.5 Ap	MNP-5 Ap	MNP-10 Ap	MNP-20 Ap
Size (nm)	263.6 ± 17.6	258.1 ± 4.6	265 ± 3.5	276.8 ± 12.5	277.7 ± 16.9
Zeta (mV)	−15.4 ± 0.6	−15.8 ± 0.2	−15.7 ± 0.7	−16.2 ± 0.3	−16.0 ± 0.4

## Data Availability

Data are contained within the article.

## References

[1] Hutchings G., Moncrieff L., Dompe C., Janowicz K., Sibiak R., Bryja A., Jankowski M., Mozdziak P., Bukowska D., Antosik P. (2020). Bone regeneration, reconstruction and use of osteogenic cells; from basic knowledge, animal models to clinical trials. J. Clin. Med..

[2] Tang Y., Wu X., Lei W., Pang L., Wan C., Shi Z., Zhao L., Nagy T.R., Peng X., Hu J. (2009). TGF-β1–induced migration of bone mesenchymal stem cells couples bone resorption with formation. Nat. Med..

[3] Paspaliaris V., Kolios G. (2019). Stem cells in osteoporosis: From biology to new therapeutic approaches. Stem Cells Int..

[4] Perez J.R., Kouroupis D., Li D.J., Best T.M., Kaplan L., Correa D. (2018). Tissue engineering and cell-based therapies for fractures and bone defects. Front. Bioeng. Biotechnol..

[5] Maciel G.B.M., Maciel R.M., Danesi C.C. (2023). Bone cells and their role in physiological remodeling. Mol. Biol. Rep..

[6] Yoshida C.A., Komori H., Maruyama Z., Miyazaki T., Kawasaki K., Furuichi T., Fukuyama R., Mori M., Yamana K., Nakamura K. (2012). SP7 inhibits osteoblast differentiation at a late stage in mice. PLoS ONE.

[7] Jing Z., Liang Z., Yang L., Du W., Yu T., Tang H., Li C., Wei W. (2022). Bone formation and bone repair: The roles and crosstalk of osteoinductive signaling pathways. Process Biochem..

[8] Schupbach D., Comeau-Gauthier M., Harvey E., Merle G. (2020). Wnt modulation in bone healing. Bone.

[9] Leucht P., Lee S., Yim N. (2019). Wnt signaling and bone regeneration: Can’t have one without the other. Biomaterials.

[10] Maeda K., Kobayashi Y., Koide M., Uehara S., Okamoto M., Ishihara A., Kayama T., Saito M., Marumo K. (2019). The regulation of bone metabolism and disorders by Wnt signaling. Int. J. Mol. Sci..

[11] Sugimoto A., Miyazaki A., Kawarabayashi K., Shono M., Akazawa Y., Hasegawa T., Ueda-Yamaguchi K., Kitamura T., Yoshizaki K., Fukumoto S. (2017). Piezo type mechanosensitive ion channel component 1 functions as a regulator of the cell fate determination of mesenchymal stem cells. Sci. Rep..

[12] Shen Y., Pan Y., Guo S., Sun L., Zhang C., Wang L. (2020). The roles of mechanosensitive ion channels and associated downstream MAPK signaling pathways in PDLC mechanotransduction. Mol. Med. Rep..

[13] Sun W., Chi S., Li Y., Ling S., Tan Y., Xu Y., Jiang F., Li J., Liu C., Zhong G. (2019). The mechanosensitive Piezo1 channel is required for bone formation. eLife.

[14] Unnithan A.R., Sasikala A.R.K., Shrestha B.K., Lincoln A., Thomson T., El Haj A.J. (2022). Remotely Actuated Magnetic Nanocarpets for Bone Tissue Engineering: Non-Invasive Modulation of Mechanosensitive Ion Channels for Enhanced Osteogenesis. Adv. Funct. Mater..

[15] Rotherham M., Nahar T., Broomhall T.J., Telling N.D., El Haj A.J. (2022). Remote magnetic actuation of cell signalling for tissue engineering. Curr. Opin. Biomed. Eng..

[16] Pankhurst Q.A., Thanh N.T.K., Jones S.K., Dobson J. (2009). Progress in applications of magnetic nanoparticles in biomedicine. J. Phys. D Appl. Phys..

[17] Hu B., Rotherham M., Farrow N., Roach P., Dobson J., El Haj A.J. (2022). Immobilization of wnt fragment peptides on magnetic nanoparticles or synthetic surfaces regulate wnt signaling kinetics. Int. J. Mol. Sci..

[18] Bonnemay L., Hoffmann C., Gueroui Z. (2015). Remote control of signaling pathways using magnetic nanoparticles, Wiley Interdiscip. Rev. Nanomed. Nanobiotechnol..

[19] Henstock J.R., Rotherham M., El Haj A.J. (2018). Magnetic ion channel activation of TREK1 in human mesenchymal stem cells using nanoparticles promotes osteogenesis in surrounding cells. J. Tissue Eng..

[20] Gonçalves A.I., Rotherham M., Markides H., Rodrigues M.T., Reis R.L., Gomes M.E., El Haj A.J. (2018). Triggering the activation of Activin A type II receptor in human adipose stem cells towards tenogenic commitment using mechanomagnetic stimulation. Nanomed. Nanotechnol. Biol. Med..

[21] Cartmell S.H., Dobson J., Verschueren S.B., El Haj A.J. (2002). Development of magnetic particle techniques for long-term culture of bone cells with intermittent mechanical activation. IEEE Trans. Nanobiosci..

[22] Rotherham M., Henstock J.R., Qutachi O., El Haj A.J. (2018). Remote regulation of magnetic particle targeted Wnt signaling for bone tissue engineering, Nanomedicine Nanotechnology. Biol. Med..

[23] Byun J. (2021). Recent progress and opportunities for nucleic acid aptamers. Life.

[24] Ramaswamy V., Monsalve A., Sautina L., Segal M.S., Dobson J., Allen J.B. (2015). DNA aptamer assembly as a vascular endothelial growth factor receptor agonist. Nucleic Acid Ther..

[25] Dobson J.P., Allen J. (2020). Magnetic particle conjugates and methods of activating cell signaling. U.S. Patent.

[26] James S., Fox J., Afsari F., Lee J., Clough S., Knight C., Ashmore J., Ashton P., Preham O., Hoogduijn M. (2015). Multiparameter analysis of human bone marrow stromal cells identifies distinct immunomodulatory and differentiation-competent subtypes. Stem Cell Rep..

[27] Saleh F., Carstairs A., Etheridge S.L., Genever P. (2016). Real-time analysis of endogenous Wnt signalling in 3D mesenchymal stromal cells. Stem Cells Int..

[28] Rotherham M., El Haj A.J. (2015). Remote activation of the Wnt/β-catenin signalling pathway using functionalised magnetic particles. PLoS ONE.

[29] Vlashi R., Zhang X., Wu M., Chen G. (2023). Wnt signaling: Essential roles in osteoblast differentiation, bone metabolism and therapeutic implications for bone and skeletal disorders. Genes Dis..

[30] Etheridge S.L., Spencer G.J., Heath D.J., Genever P.G. (2004). Expression profiling and functional analysis of wnt signaling mechanisms in mesenchymal stem cells. Stem Cells.

[31] Lojk J., Marc J. (2021). Roles of non-canonical Wnt signalling pathways in bone biology. Int. J. Mol. Sci..

[32] Saal H.M., Prows C.A., Guerreiro I., Donlin M., Knudson L., Sund K.L., Chang C.-F., Brugmann S.A., Stottmann R.W. (2015). A mutation in FRIZZLED2 impairs Wnt signaling and causes autosomal dominant omodysplasia. Hum. Mol. Genet..

[33] Liang D., Wang X., Mittal A., Dhiman S., Hou S.-Y., Degenhardt K., Astrof S. (2014). Mesodermal expression of integrin α5β1 regulates neural crest development and cardiovascular morphogenesis. Dev. Biol..

[34] Wang L., Zheng F., Song R., Zhuang L., Yang M., Suo J., Li L. (2022). Integrins in the regulation of mesenchymal stem cell differentiation by mechanical signals. Stem Cell Rev. Rep..

[35] Liu C., Kaneko S., Soma K. (2008). Expression of integrinα5β1, focal adhesion kinase and integrin-linked kinase in rat condylar cartilage during mandibular lateral displacement. Arch. Oral Biol..

[36] McIlhenny S.E., Hager E.S., Grabo D.J., DiMatteo C., Shapiro I.M., Tulenko T.N., DiMuzio P.J. (2010). Linear shear conditioning improves vascular graft retention of adipose-derived stem cells by upregulation of the α5β1 integrin. Tissue Eng. Part A.

[37] Wang N. (2017). Instant integrin mechanosensing. Nat. Mater..

[38] Kong F., García A.J., Mould A.P., Humphries M.J., Zhu C. (2009). Demonstration of catch bonds between an integrin and its ligand. J. Cell Biol..

[39] Fromigué O., Brun J., Marty C., Da Nascimento S., Sonnet P., Marie P.J. (2012). Peptide-based activation of alpha5 integrin for promoting osteogenesis. J. Cell. Biochem..

[40] Sonowal H., Kumar A., Bhattacharyya J., Gogoi P.K., Jaganathan B.G. (2013). Inhibition of actin polymerization decreases osteogeneic differentiation of mesenchymal stem cells through p38 MAPK pathway. J. Biomed. Sci..

[41] Du J., Zu Y., Li J., Du S., Xu Y., Zhang L., Jiang L., Wang Z., Chien S., Yang C. (2016). Extracellular matrix stiffness dictates Wnt expression through integrin pathway. Sci. Rep..

[42] Subjakova V., Oravczova V., Hianik T. (2021). Polymer nanoparticles and nanomotors modified by DNA/RNA aptamers and antibodies in targeted therapy of cancer. Polymers.

[43] Zhang Y., Lai B.S., Juhas M. (2019). Recent advances in aptamer discovery and applications. Molecules.

[44] Li K., Liu S., Li J., Yi D., Shao D., Hu T., Zheng X. (2023). Manganese supplementation of orthopedic implants: A new strategy for enhancing integrin-mediated cellular responses. Biomater. Sci..

[45] Giudice V., Mensitieri F., Izzo V., Filippelli A., Selleri C. (2020). Aptamers and antisense oligonucleotides for diagnosis and treatment of hematological diseases. Int. J. Mol. Sci..

[46] Jones E., McGonagle D. (2008). Human bone marrow mesenchymal stem cells in vivo. Rheumatology.

[47] Mildmay-White A., Khan W. (2017). Cell surface markers on adipose-derived stem cells: A systematic review. Curr. Stem Cell Res. Ther..

[48] Huang W., Yang S., Shao J., Li Y.-P. (2007). Signaling and transcriptional regulation in osteoblast commitment and differentiation. Front. Biosci. J. Virtual Libr..

[49] Marupanthorn K., Tantrawatpan C., Kheolamai P., Tantikanlayaporn D., Manochantr S. (2021). MicroRNA treatment modulates osteogenic differentiation potential of mesenchymal stem cells derived from human chorion and placenta. Sci. Rep..

[50] Wülfing C., Dovedi S.J. (2023). For optimal antibody effectiveness, sometimes less is more. Nature.

[51] Hajiali H., Shahgasempour S., Naimi-Jamal M.R., Peirovi H. (2011). Electrospun PGA/gelatin nanofibrous scaffolds and their potential application in vascular tissue engineering. Int. J. Nanomed..

[52] Kilian K.A., Mrksich M. (2012). Directing stem cell fate by controlling the affinity and density of ligand–receptor interactions at the biomaterials interface. Angew. Chem. Int. Ed..

[53] Mann B.K., Tsai A.T., Scott-Burden T., West J.L. (1999). Modification of surfaces with cell adhesion peptides alters extracellular matrix deposition. Biomaterials.

[54] Neff J.A., Tresco P.A., Caldwell K.D. (1999). Surface modification for controlled studies of cell–ligand interactions. Biomaterials.

[55] Palecek S.P., Loftus J.C., Ginsberg M.H., Lauffenburger D.A., Horwitz A.F. (1997). Integrin-ligand binding properties govern cell migration speed through cell-substratum adhesiveness. Nature.

[56] Hlavacek W.S., Posner R.G., Perelson A.S. (1999). Steric effects on multivalent ligand-receptor binding: Exclusion of ligand sites by bound cell surface receptors. Biophys. J..

[57] Kilinc D., Dennis C.L., Lee G.U. (2016). Bio-Nano-Magnetic Materials for Localized Mechanochemical Stimulation of Cell Growth and Death. Adv. Mater..

[58] Evans A., Calderwood D.A. (2007). Forces and bond dynamics in cell adhesion. Science.

[59] Na S., Collin O., Chowdhury F., Tay B., Ouyang M., Wang Y., Wang N. (2008). Rapid signal transduction in living cells is a unique feature of mechanotransduction. Proc. Natl. Acad. Sci. USA.

